# Measuring nationwide US online media mentions of fish found in the North Central Region

**DOI:** 10.1371/journal.pone.0356559

**Published:** 2026-08-18

**Authors:** Michael L. Smith, Kwamena Quagrainie, Nicole Olynk Widmar

**Affiliations:** Purdue University Department of Agricultural Economics, West Lafayette, Indiana, United States of America; Benha University, EGYPT

## Abstract

This paper focuses on quantifying online media discussions of fish species that are associated with recreational fishing cultures in the United States. Specifically, we observe online and social media discussions of Bluegill, Largemouth Bass, Walleye, Rainbow Trout, and Yellow Perch, which are major fishes in the North Central Region. Observations were drawn from social and news media posted online in the US between July 1^st^, 2020, and July 1^st^, 2024. A total of 611,052 social media mentions of the fish species were found. We also investigate the relationship between national online media mentions and net sentiment to the surface temperatures recorded in the Great Lakes using pairwise correlations. We find consistent levels of mentions of these species. We sub-search within these results, looking for discussions specific to the origin of the fish -- wild caught or farmed. We find only 1% to 5% of mentions of each species specifically mentioned wild or farmed origins. We evaluate the net sentiment of the mentions of the fish, finding generally positive sentiment. Mentions of these fish are generally more significantly correlated (than net sentiment) to surface temperatures in the Great Lakes region. These findings suggest that these fish were referred to in general terms, and seldom in terms of whether they were caught or farmed. The results between surface temperatures and mentions highlight the importance of careful consideration of catchability and temperature levels in fisheries management.

## Introduction

Social listening is increasingly being used to assess perceptions, preferences, and emotions about products and services. An emerging body of literature finds that online media data reflects seasonal patterns of nature-based recreational activity [1]. Quantifying online media data, including net sentiment of posted media, may be informative for recreational fisheries managers, as the information may be reflective of stakeholder opinions on various fishery issues. In addition to stakeholder feelings, online media data may contain useful signals for policy makers and wildlife managers, including species health and presence, and recreational visitation patterns [2]. In the North Central Region (NCR), thousands of lakes support several fish species with cultural, recreational, and economic importance to the region. Studies from creel surveys of anglers – in which fisheries managers interview anglers to understand their activities, including species being harvested and catch rates (among other data) – in the region have found that popular species among anglers include Bluegill (Lepomis macrochirus), Largemouth Bass (Micropterus nigricans), Walleye (Sander vitreus), Yellow Perch (Perca flavescens), and Rainbow Trout (Oncorhynchus mykiss) [3–6]. While Bluegill, Largemouth Bass, Walleye, and Yellow Perch are native to the NCR, Rainbow Trout is an introduced (non-native) species [5,68]. These species, herein referred to as NCR fishes of the U.S., are major fish (wild and farmed) utilized as both food fish and recreational fish.

In addition to recreational purposes, the market for these species of fish is large. For farmed fishes, the 2023 USDA Census of Aquaculture reported that the value of aquaculture products sold in the NCR increased by about 66% from a reported $42.7 million in 2018 to $71.1 million in 2023 [7]. For farmed Yellow Perch and Trout sold as food fish, the value of sales declined by 85% and 25%, respectively, from 2018 to 2023. Both farmed Yellow Perch and Trout are also considered recreational fishes. USDA [7] reports an increase in the number of farmed Trout produced and distributed for conservation, recreation, enhancement, or restoration purposes in the NCR from 15 million fish to 21 million fish, a 38% increase. The sales value of the other farmed fishes increased from 2018 to 2023, including Largemouth Bass sold as sportfish (15%), Bluegill (20%), and Walleye as sportfish (42%). It suggests a possible shifting or substitution of farmed food fish sales to recreational sales for some of these fishes. A substantial portion of sport fish sales in the NCR is farmed Largemouth Bass, accounting for about 53% in 2018 and 43% total sport fish production in 2023. Largemouth Bass is followed by Walleye; Walleye accounted for 33% in both 2018 and 2023. Some sport fish from the NCR are also featured through ethnic food markets.

The purpose of this study is to understand public sentiment towards the five fishes and to explore how this data may be useful to fisheries managers. Specifically, we aim to uncover the ‘what’ and the ‘why’ of opinions and views expressed online by members of the general public. Through online posts, users express their feelings and opinions through agreeing or disagreeing, satisfaction or dissatisfaction, and their interpretation of various topics or events, including fisheries [8] and wildlife [9]. This is relevant in the field of human dimension studies on human behavior, beliefs, and attitudes relating to fisheries because of information exchange and opinions expressed online. An understanding of the values and preferences for the NCR fishes contributes to studies on angler satisfaction from catch-related factors such as catch rate, size of caught fish, and fish harvest [10,11].

Although anglers can control some aspects of their fishing practices, they have limited ability to manage the water temperatures fish are exposed to during capture [12]. Meyer et al [12] found that higher water temperatures may naturally reduce angler catch rates for stream-dwelling salmonids. Another recent study indicated the need to consider river temperatures when evaluating fish catchability because, as water temperatures continue to rise, avoiding catch-and-release during periods of thermal stress and adopting best handling practices will become increasingly important for sustaining recreational fisheries [13]. We also examine correlations among nationwide mentions of the NCR fishes and surface water temperatures of the Great Lakes. Some of the comments online may originate in US locations not near the Great Lakes. As such, the relationship (and subsequent interpretation) between online media collected here and the Great Lakes surface temperatures reflects a proof of concept and directional movement in season and sentiment rather than a tightly knit causal relationship.

The web and social media provide ample data that can be leveraged to guide policy decisions in wildlife and fisheries management [14]. For example, Vacura, Camp & Venturelli [8] use angler online posts on Walleye to validate harvest management and governance in 9 U.S. states to study the factors that drive Walleye angler satisfaction. Fidino, Herr & Magle [9] found that analyzing online comments on coyote, opossum, and raccoon represented a useful analytical approach to studying people’s beliefs about wildlife. Ellman et al. [15] employed online and social media analysis to study people’s beliefs about pollinators. The use of online data analytics is relatively cheaper in terms of costs compared to traditional survey methods, including creel surveys [8,14]. These survey techniques are useful to understand large-scale social and ecological interactions but suffer from a disconnect between survey methods and the questions posed by researchers who may not comprehensively understand the social or ecological dimension [16]. Survey research, including creel surveys, also requires time- and place-specific, on-site administration, which generally requires sampling efforts to persist across days and locations to ensure the interview panel is adequately sized [16–18]. Related methods include mail, phone, and license-based survey techniques. These, along with creel surveys, may be very useful to derive anglers’ satisfaction, preferences (environmental, infrastructure, and regulatory), and willingness-to-pay for fishing experiences [19,20]. Many of these specific dimensions are strengths of survey research that online media cannot adequately perform. Contrasting this, online media listening can be continuous with more automation for analysis built out before (or as) data collection commences [21]. With online media, real-time analysis is attractive because it allows researchers to gauge the time and extent to which a topic injects itself into public discussion (and if applicable, when the discussion moves on from a subject). However, there are challenges posed by the volume of data to be collected (as there may be thousands, if not millions, of posts on a topic) and in differentiating true public interest from algorithm-driven activity including bots, hype, and viral occurrences [22]. The lack of questioning or prompting for responses on a given topic is often an attractive aspect of social and online media analysis, compared to surveying [23]. Other limitations of online media data include data access and privacy concerns [24], which commercial platforms may assist users in overcoming at the expense of replicability [25]. Online media listening positions in the context of these methods by offering a cost-efficient, continuous, high-volume alternative to more traditional survey-based research in select applications [26].

We apply sentiment analysis to online media data to quantify public attitudes that could inform management and regulatory processes. Trends in sentiment may offer an indicator of how specific factors relate to people’s views of the recreational fisheries sector, which may warrant further investigation, possibly using alternative methods. If validated against established methods, sentiment analysis may also assist resource managers in identifying areas of concern that could inform future management decisions and interventions. Existing research has found that online media sentiment (and corresponding counts of mentions) behaves differently across topics. For example, crisis topics tend to see time-sensitive mention surges combined with negative sentiment [27]. Contrasting this, community-based topics surrounding recreation and sport may see online media sentiment and mentions behavior more in line with uses and gratification theory [28]. The most similar example being a recent study of online media use in birdwatching communities, which finds that users tend to seek information and gratification through status signals, which may in turn promote responsible birdwatching behavior [28]. By tracking mentions and sentiment over time, this approach may reveal potential issues [29,30], complaints or concerns [31,32], or market trends [33,34], that could complement existing inputs to policy and regulatory discussions.

By collecting social and news media posts about these species, this paper builds upon previous inquiries into online media discussion in natural resources [8,9,14]. Harnessing online media information allows for open dialogue among stakeholders and promotes future research, analysis, and collaboration within a sector. The overarching goal is to explore the volume of online discussion and sentiment towards the NCR fishes and their trends (positive or negative) over time.

This study contains four research questions. First, what are the discussion volume and temporal trends of online media mentions of the NCR fish species (Bluegill, Largemouth Bass, Walleye, Yellow Perch, and Rainbow Trout) across the four years of study? Second, how does public sentiment of these discussions differ between the species and with respect to the origin of the fish species (wild or farmed), and what are the common themes of these discussions? Third, how are mention volumes and associated sentiment scores (obtained from mentions originating nationwide) correlated with surface water temperatures in the Great Lakes region, consistent with the well-established thermal ecology preferences of these species? Lastly, what use are commercial social listening platforms as a complementary approach to traditional creel surveys, which monitor public attitudes towards recreational fisheries? This paper presents a proof of concept. Due to the novelty of this approach in this context, we lack strong priors to make predictions. We intend to be descriptive in nature, while drawing on prior literature to motivate directional expectations where possible.

Due to the novelty of this method in the context of human-fish interaction, we evaluate the usefulness of online media collection to understand angler and public sentiment. This is accomplished through the application of sentiment analysis to assess online opinions and emotions on NCR fishes utilizing online listening tools and natural language processing. We then quantify online and social media sentiment in terms of positivity and negativity associated with the NCR fishes over time – farmed and wild-caught. We categorize online content and highlight patterns of how people perceive the food fish and sport fish sectors, and assess implications of online media for recreational fishing. These findings help us to better understand the contexts in which these fishes are discussed, as well as common applications of these fish (once caught or harvested).

The study also examines nationwide online media data by interacting findings with data on surface temperatures in the Great Lakes and St. Clair River. This approach links aggregate nationwide US online discussion volume in the fishes to the environmental conditions in the NCR where these fish play important roles both ecologically and recreationally. We cannot assume a linkage between these mentions of the fish, which occur nationwide, and the Great Lakes temperature but given their prominence in the region, we include this analysis as a proof of concept for online media listening data interacted with ecological data.

While some mentions may originate beyond the likely geography of anglers in the North Central region, we maintain that these insights may still be informative. These results yield a broad national-level attention as opposed to localized angler attention. This yields a better understanding of natural dynamics that influence these discussions and emotions, and also the extent to which online media insights can be paired with readily available secondary data on environmental factors. We therefore contribute an exploratory assessment of whether large-scale environmental signals can reflect in aggregated online discourse occurring contemporaneously.

## Materials and methods

This paper employs social media listening tools to collect and analyze a novel dataset that quantifies the volume and sentiment of online and social media posts about NCR fishes. We develop the dataset using the Quid platform (formerly known as Netbase) to scrape various online sources for mentions of NCR species [35]. Our collection fully complied with the terms and conditions maintained by each platform from which data is collected (further, we are in full compliance with the terms and conditions required by Quid) [35]. This research is not subject to IRB approval as it does not contain personally identifiable information on any subjects, and all data collected was publicly available. Quid’s scraping process enables researchers to parameterize a search based on a series of included and excluded terms, geographies, and to craft a series of sub-searches within the total group of observed data [35].

**Fig 1:**
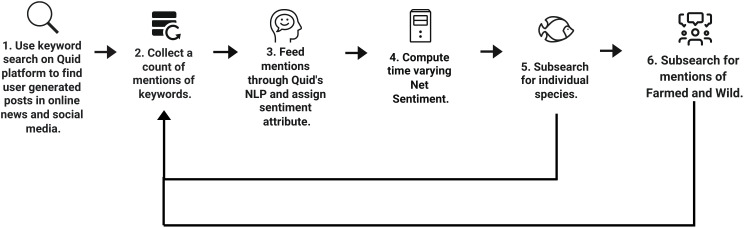
A visualization of the data collection process.

Quid analyzes posts to quantify the number of mentions of included terms in the dataset [36]. Mentions are generally subsets of posts specific to an included term, and there may be more than one included term mentioned in a single post [37]. As such, the number of mentions is generally larger than the number of posts. Henceforth, we analyze counts of mentions. In this study, we sub-search the total set of results, which allowed us to isolate and analyze mentions of each of the NCR species and mentions of the species in the context of “farmed” and “wild”, following a methodology using themes that have been developed through prior works, including [34,38,39]. We are then able to compare data between the species, and to compare how public perceptions differ based on the context of wild-caught or farmed origins (example: Yellow Perch Wild vs Yellow Perch Farmed). Our process is visualized in Fig 1. Domains used to collect mentions include: twitter.com, hotspotoutdoors.com, express-press-release.net, kchanews.com, airgunmaniac.com, iceshanty.com, opentable.com, forums.fishusa.com, kiow.com, 24hourcampfire.com, boards.4channel.org, mdpi.com, newsbreak.com, targetwalleye.com, ncesc.com, texasfishingforum.com, madison.com, trapperman.com, walleyecentral.com, and bbcboards.net. 73% of mentions are observed on Twitter.

Social media listening is a useful method of data collection because it enables researchers to capture public opinion in a manner that is unprompted by the researcher, mitigating potential biases that researchers may induce in survey and experimental methods [8,9,14,30]. Quid has been used in the peer review context to study topics related to food and health [15,29,31,33,37,39–41], recreation [32,38], and less tangible subjects of online discourse such as investments, and seasonal trends [32,34,42–45]. Quid randomly samples 10% of collected mentions for sentiment scoring (positive, negative, or neutral), a platform default designed to produce a representative estimate of sentiment across the full collection of mentions collected [36]. This sentiment analysis focuses specifically on how the keyword primary terms are used. During query development, the authors conducted an iterative manual review of samples of positive, negative, and neutral posts to assess the validity of keyword and sentiment classifications, accounting for excludable results such as homonyms, unrelated slang, and sentiment scoring. Net sentiment is calculated as the net of positive and negative sentiments as a percentage of the total of the two sentiments; therefore, the net sentiment score is bounded between +100% and −100% [35,37,46]. Effectively, Quid’s net sentiment offers an exploratory estimate of directionality (positive to negative) of online discussions. Due to limitations posed by the proprietary nature of the Quid platform, we treat these net sentiment scores as directional indicators showing whether the emotional valence of the discussion is more positive or negative, instead of a specific and accurate measurement of the extent to which conversations are positive or negative.

Posts (and specifically the mentions they contain) were collected if they originated in the US between July 1^st^, 2020, and July 1^st^, 2024, parameterizing a 4-year study period. Data presented for the years 2020 and 2024 are limited to the period of observation; as such, these reflect partial years of observation. We present data on a weekly basis (210 weeks), resulting in a total of 737,227 mentions for NCR fishes. Combining net sentiment scores with the volume of mentions, we analyze two time-varying data points collected.

The weekly mentions and weekly net sentiment are presented for each of our species, as well as the sub-searches that delineate each species for “wild” and “farmed”. To further analyze the data, we analyze the data for autocorrelation and stationarity using the Ljung-Box Portmanteau test for white noise and the Dickey-Fuller unit root test [47,48]. We find that the time series exhibits serial dependence, so we do not interpret correlations as independent-sample measures of association. We instead rely on Spearman rank correlations as a non-parametric and robust approach to measure monotonic co-movements occurring between variables [49,50]. This approach is less sensitive to non-normality and outliers than Pearson correlations would be. We note that this does not eliminate autocorrelation; our diagnostics indicate short memory autocorrelation in our time series, which is consistent with prior work using correlation analysis on autocorrelated ecological time series [51] and significance testing under autocorrelation [52]. This motivates our descriptive correlation framework (as opposed to a causal inference model). We interpret correlation results as descriptive associations in a time-dependent series rather than independent cross-sectional relationships [53]. Mentions are further analyzed to determine the top terms commonly used alongside the terms we specifically search for. These top terms are useful to provide context for the ways that users talked about the fishes.

We extend this analysis by including data on the surface temperatures of water in the Great Lakes (Erie, Ontario, Huron, Superior, and Michigan, along with St. Clair, which connects Huron and Erie) [54]. We analyze the relationship between US nationwide online media data (mention counts and net sentiment) for each of the fishes with average Great Lakes Surface Environmental Analysis (GLSEA) surface water temperature, all observed on a daily basis, aggregated into weekly average mean temperature. Using these data series, we estimate Spearman rank correlations.

## Results and discussion

Annual descriptive statistics, which show weekly averages and standard deviations for mentions and net sentiment, are presented in Table 1. The results show that, over the 4-year study period, all species had a weekly average net sentiment of over +60% except wild Largemouth Bass (+37%) and wild Yellow Perch (+36%). However, the weekly average for farmed Largemouth Bass was 84% while farmed Yellow Perched had an average of 67%. The net sentiment for the two species was not consistently high over the 4-year study period relative to the other species.

**Table 1:** Descriptive statistics on weekly average mentions and net sentiment (standard deviation in parenthesis) by year.

	Mentions						Net Sentiment					
	2020	2021	2022	2023	2024	Total	2020	2021	2022	2023	2024	Total
Bluegill Total	660.4	536.5	474.6	552.5	610.1	550.5	66.6	73.9	68.2	66.0	68.7	68.8
	(383.8)	(256.6)	(195.1)	(221.2)	(299.1)	(267.0)	(17.6)	(11.8)	(18.8)	(18.6)	(19.9)	(17.3)
Bluegill Farmed	9.9	9.8	4.8	6.3	6.0	7.2	86.6	81.1	76.7	61.1	45.4	71.2
	(13.4)	(13.5)	(5.8)	(6.8)	(5.0)	(7.1)	(19.7)	(5.3)	(6.6)	(16.8)	(3.6)	(16.4)
Bluegill Wild	2.8	3.8	3.4	4.2	5.0	3.8	92.6	89.1	83.2	52.3	82.0	78.0
	(2.6)	(4.5)	(3.7)	(4.0)	(7.4)	(3.9)	(26.7)	(8.3)	(5.8)	(11.0)	(12.6)	(18.4)
Largemouth Bass Total	637.4	405.2	442.3	512.3	547.4	487.3	88.9	66.3	68.9	63.5	67.4	68.9
	(303.8)	(202.4)	(360.7)	(178.4)	(222.1)	(477.8)	(9.8)	(15.6)	(19.0)	(18.9)	(14.5)	(18.3)
Largemouth Bass Farmed	5.4	6.6	3.2	5.0	7.2	5.2	96.3	76.0	75.8	90.3	93.5	84.4
	(7.3)	(15.5)	(3.9)	(4.5)	(4.8)	(5.1)	(19.2)	(16.3)	(10.6)	(5.4)	(2.5)	(13.6)
Largemouth Bass Wild	1.9	2.8	3.4	2.9	22.0	5.1	74.1	18.8	6.7	53.3	60.2	36.8
	(2.7)	(3.5)	(4.3)	(2.9)	(82.2)	(5.2)	(44.7)	(54.9)	(28.0)	(25.5)	(3.9)	(44.6)
Walleye Total	1581.8	1629.5	1688.5	1790.5	2206.2	1745.2	63.8	65.0	54.9	64.6	64.9	62.2
	(554.9)	(479.3)	(730.4)	(540.8)	(517.6)	(1755.1)	(10.7)	(10.4)	(26.1)	(15.1)	(10.7)	(17.4)
Walleye Farmed	4.1	8.7	8.4	5.3	10.0	7.4	54.0	35.8	68.2	74.9	66.6	59.8
	(5.7)	(18.4)	(12.8)	(6.5)	(7.5)	(7.5)	(28.1)	(12.0)	(15.6)	(8.3)	(8.5)	(19.0)
Walleye Wild	11.3	16.9	26.1	20.9	34.1	21.5	78.3	72.6	70.3	42.2	66.5	64.4
	(11.4)	(21.9)	(37.6)	(23.2)	(15.1)	(21.8)	(19.4)	(14.4)	(23.4)	(16.9)	(13.2)	(21.6)
Yellow Perch Total	102.8	111.6	121.8	131.0	187.4	126.7	66.8	65.5	53.1	59.2	58.8	60.2
	(44.1)	(42.9)	(41.6)	(40.8)	(74.6)	(128.0)	(20.9)	(27.7)	(27.9)	(27.7)	(16.7)	(26.0)
Yellow Perch Farmed	0.6	0.9	1.5	0.7	0.7	0.9	0.0	67.7	72.3	77.2	100.0	66.6
	(0.9)	(2.2)	(3.4)	(2.6)	(1.0)	(1.0)	(0.0)	(24.2)	(19.5)	(23.4)	(0.0)	(31.7)
Yellow Perch Wild	1.3	1.2	1.8	1.3	1.5	1.4	66.7	11.4	14.6	32.5	100.0	35.9
	(3.9)	(2.2)	(2.7)	(2.1)	(1.8)	(1.5)	(48.0)	(43.7)	(24.2)	(55.5)	(0.0)	(51.0)

Numbers are the weekly average with standard deviation in parentheses. 2020 and 2024 are partial years, observing July-December 2020 and January-June 2024.

Broadly speaking, this suggests the disposition (positive, negative, or neutral) of mentions was mostly positive for these species.

We then compare data by species to garner insights in a few ways. First, we examine levels of weekly mention counts by species, which are rather low relative to many social listening studies focusing on food-related issues and topics related to environmental attributes [15,31,40–42,55]. We also examine the weekly positivity vs negativity associated with each species by examining the net sentiment by species. All species had 1,000 or fewer mentions in 200 weeks out of the 210-week study period, except Walleye, which had 1,000 or fewer mentions in 6 weeks, 1,001–2,000 mentions in 143 weeks, and 2,001–3000 mentions in 55 weeks. Meanwhile, Bluegill, Rainbow Trout, and Largemouth Bass had mentions of 1,001–2,000, respectively, in 9, 8, and 7 weeks of the study period. Walleye also had mentions of over 3,001–4,000 in 5 weeks of the study period. Each species had positive net sentiment throughout the 210 weeks of study, with over 50% scores in more than half of the study period. For example, Bluegill had net sentiment scores of above 50% in 183 weeks out of the 210-week study period, followed by Rainbow Trout, 180 weeks, Walleye, 178 weeks, Largemouth Bass, 177 weeks, and Yellow Perch, 147 weeks.

We also present results by their respective sub-searches for mentions specific to farming and wild caught, and extract insights on the top terms mentioned, which serve as a basis to discuss the context in which the fishes are mentioned.

### Walleye

**Fig 2:**
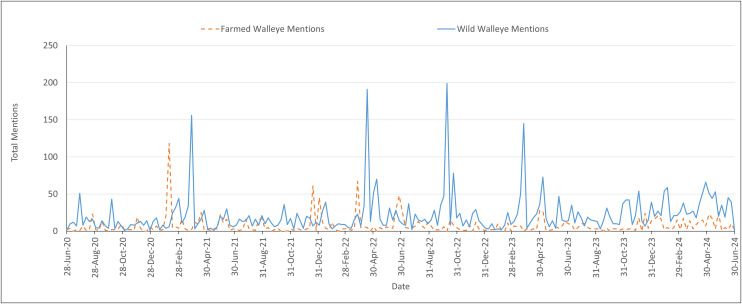
Walleye weekly mentions.

As noted, Walleye is the most popular among the species in terms of the absolute number of mentions. While weekly mentions of the other species were in their 10s and 100s, Walleye weekly mentions were in the 1000s, averaging 1,582, 1,630, 1,689, 1,791, and 2,206, respectively, in 2020, 2021, 2022, 2023, and 2024 (Fig 2). The annual weekly average is roughly 1,745 per week, and net sentiment averaged 62% weekly. Walleye support harvest-oriented recreational fisheries in the NCR, with various state Departments of Natural Resources supporting the industry with stocking from aquaculture production to supplement natural reproduction [56,57]. The 2023 USDA Census of Aquaculture shows Walleye were the second most stocked fish in the United States, behind Rainbow Trout [7].

**Fig 3:**
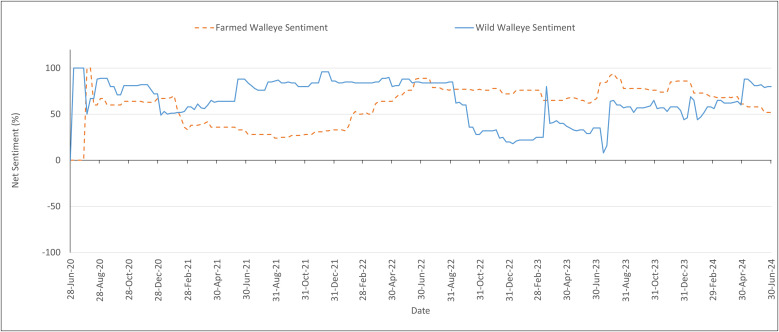
Walleye weekly net sentiment.

The magnitude of the rise in average weekly mentions was even higher in walleye’s subcategories, with farmed Walleye seeing a 140% increase from 2020–2024 and wild seeing a 200% increase (Fig 3A). Spikes in mentions are observed around the summer months when these fish are in season. The net sentiment of media mentioning Walleye was generally positive, and there is a difference in net sentiment between mentions of wild and farmed Walleye. Though total net sentiment remained stable throughout the study period, wild average weekly net sentiment fell 15% from 2020 to 2024. Farmed walleye saw a 23% increase in net sentiment between 2020 and 2024, with a weekly average net sentiment over the period of observation at 60%, as shown in Fig 3.

Neither wild-caught nor farmed Walleye was frequently discussed in a negative light. Walleye were commonly discussed in the context of fishing for them, and attributes related to the catch (such as a nice catch or great walleye) were common. Fishing tournaments were also frequently mentioned in the context of walleye, including a controversial fish-stuffing incident at a Lake Erie fishing tournament [58]. Another common topic of discussion related to Walleye was eating. This was not always positive; a term frequently used to describe Walleye was ‘mushy’.

### Bluegill

**Fig 4:**
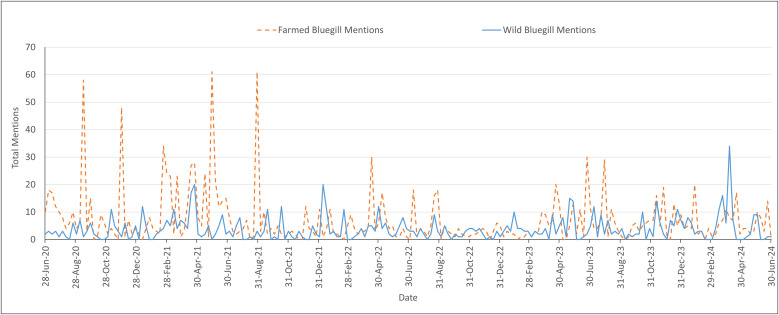
Bluegill mentions.

**Fig 5:**
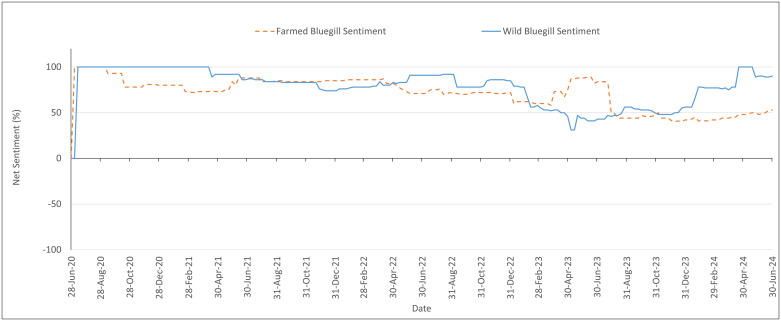
Bluegill weekly net sentiment.

Bluegill net sentiment was mostly positive and fairly stable across the period of study. Net sentiment is mostly positive and relatively stable, though the trend shows weekly variability of total net sentiment over the period (Fig 4). Overall, we found Bluegill averaged 550 total mentions per week, with a net sentiment averaging +69% per week (Fig 5). Discussion of Bluegill on social media is consistent throughout the year and generally increases around summer months, particularly for wild Bluegill, as shown in Fig 4A. Most mentions of wild Bluegill were about 10 or fewer per week in 191 weeks of the 210-week study period compared to 167 weeks of mentions for farmed Bluegill, which saw higher numbers of mentions in 2020 and 2021, the spring of 2022, and the summer of 2023. The search results analyzed were mostly positive in net sentiment for both farmed Bluegill and wild Bluegill. This suggests that Bluegill is generally discussed positively regardless of its source (farmed or wild). There were a few mentions of wild and farmed sources, making it difficult to derive many insights from these sub-searches. Net sentiment of wild Bluegill was positive throughout data collection, averaging +78% over the weeks observed. Farmed sentiment declined from an average of +95% at the start to an average of +60% in the final months of observation, generally trending downwards over the 4 years (Figure).

When Bluegill are discussed, it often occurs in similar contexts over the years of observation. Commenters frequently mentioned ‘fishing’ and reported ‘catching’ ‘nice bluegill’. There was also frequent mention of the pace of fishing bluegill being ‘slow’ across the 4 years of observation. In a study of the social vulnerability of Bluegill, Louison et al [59] concluded that Bluegill are generally more social and more vulnerable to capture by anglers. The study asserts that angling constitutes a mortality risk to Bluegill, and their social nature predisposes them to a greater risk of catching [59].

### Largemouth Bass

**Fig 6:**
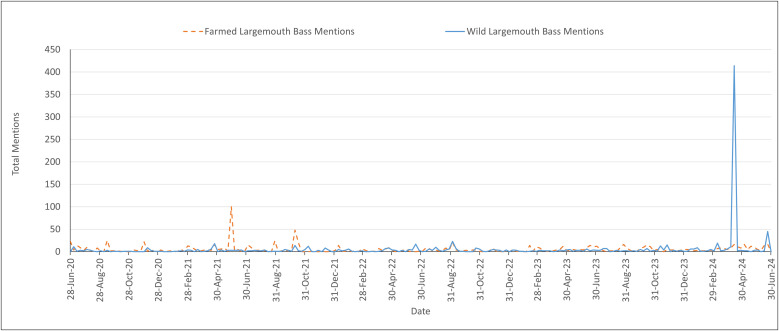
Largemouth bass weekly mentions.

**Fig 7:**
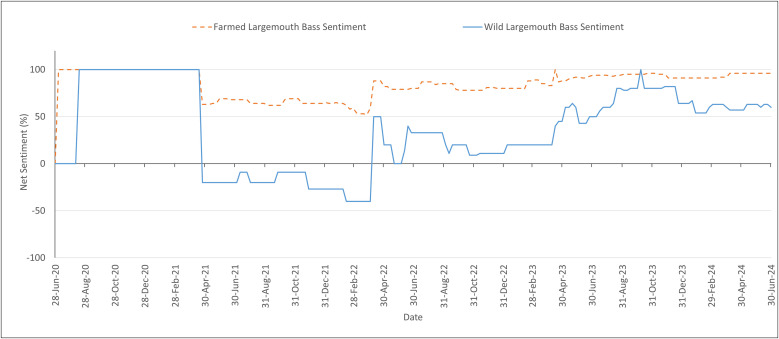
Largemouth bass weekly net sentiment.

Mentions of Largemouth Bass were consistent throughout the period of data collection, averaging roughly 487 per week with a standard deviation of 264 mentions per week (Table 1). As Figs 6 and 7 show, mentions and net sentiment in farmed and wild largemouth bass were stable around 5 mentions per week in each group, with net sentiment showing that farmed (+84%) was more well-liked than wild (+37%). While Largemouth Bass farmed and wild discussions were also sparse, compared to the overall discussion of the fish, this may reflect the angling practices in the upper Midwest, where nearly all Largemouth Bass caught are released by anglers [4,6]. The prevalence of discussions regarding the difficulty catching Largemouth Bass is more than that of any other species included in this paper.

Largemouth Bass net sentiment was mostly positive in the total collection of mentions of this species (Fig 5B). While total net sentiment remained positive and fairly consistent, farmed Largemouth Bass largely had high positive sentiment, with a few weeks showing 100% (positive) sentiment in 2020 (Fig 5B). Among the five species, it had the highest annual average of 84% (Table 1). Wild Largemouth also had higher values in 2020, but sentiment declined from the week of April 25^th^, 2021, into 2022. By April of 2022, the net sentiment of Wild Largemouth Bass was > 0, indicating a higher proportion of positive mentions compared to negative mentions.

Conversations regarding the Largemouth Bass commonly entail discussion of catching and finding use for these fish, including meals. In 2021, the commenters frequently mentioned the abundance of Largemouth Bass. Some commentators reported difficulties catching Largemouth Bass, and bait was a common topic of discussion in Largemouth Bass but not in other species. Difficulties catching the Largemouth Bass are not surprising. Sullivan et al. (2020) suggest that climate change is pushing populations of warm-water fish like Largemouth Bass towards lakes in the northern edge of the US, leading to northward expansion, which makes such species sensitive to fishing mortality.

### Rainbow Trout

**Fig 8:**
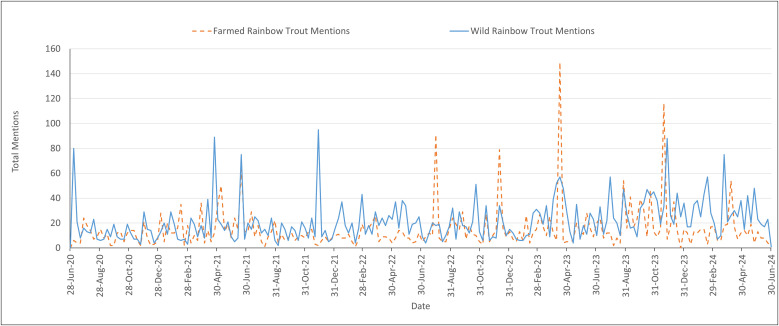
Rainbow trout weekly mentions.

**Fig 9:**
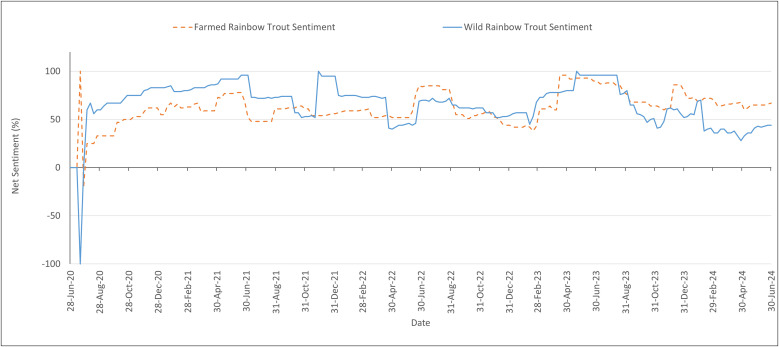
Rainbow trout weekly net sentiment.

Over the period of data collection, Rainbow Trout saw average weekly mentions of 577 and a standard deviation of 540 mentions per week (Table 1). As seen in Fig 6A, average weekly mentions of both farmed and wild Rainbow Trout showed an increasing trend from 2020 to 2024, led primarily by wild mentions (100%), while farmed saw a 46% increase from 2020 to 2024 (Fig 8). Overall, Rainbow Trout net sentiment rose 7% from 2020 to 2024, but wild Rainbow Trout saw a 14% fall in net sentiment over those four years. Farmed Rainbow Trout average weekly net sentiment rose 46% from 2020 to 2024 (Fig 9).

When discussing Rainbow Trout, people commonly referred to the practice of fishing for them. Mentions of Rainbow Trout included common discussions of cooking attributes like fresh, dry, and taste. The top terms associated with Rainbow Trout reinforce the fishing and eating relationships, the most common terms being catch, eat, and order. Rainbow trout was criticized in the mentions for anatomical issues (including skeletal deformities) and its nutritional value.

### Yellow Perch

**Fig 10:**
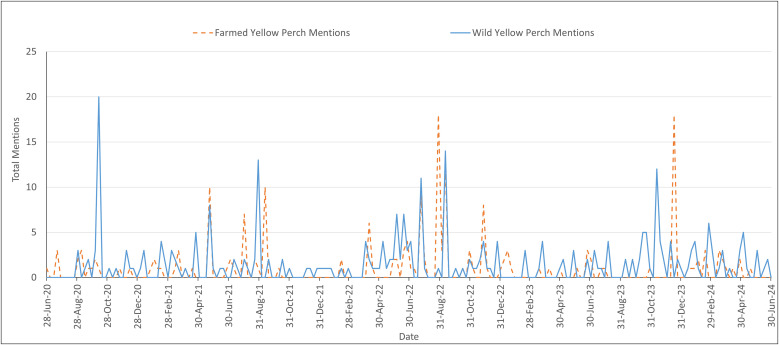
Yellow perch weekly mentions.

**Fig 11:**
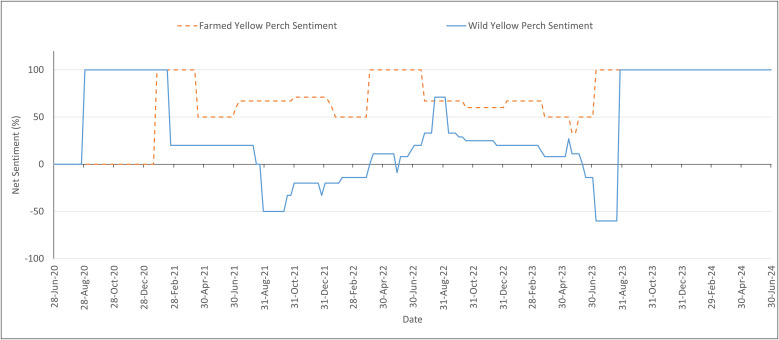
Yellow perch weekly net sentiment.

Yellow Perch averaged 127 mentions per week over the period of observation, with a standard deviation of 53 (Table 1). Yellow Perch is a cool-water fish and is active even during the winter months under lake ice, providing anglers with fishing opportunities throughout the year. They can be found in Lake Erie, Lake St. Clair, Saginaw Bay, the eastern end of the Upper Peninsula, and southern Michigan, among other places around the US [60]. Mentions of Yellow Perch were less common than other species observed in this study, averaging around 126 per week, and net sentiment averages 60% per week (Table 1). Between 2020 and 2024, we saw the average weekly total mentions of farmed Yellow Perch rise by 21% and wild Yellow Perch rise by 14% (Fig 10). However, these two subcategories barely achieved an average of 1 mention per week in any year. Over the four years, the average weekly net sentiment of Yellow Perch declines from +67 to +59, but the net sentiment of mentions remains mostly positive at +60% (Fig 11). Farmed Yellow Perch sentiment remains positive throughout the collection, but for wild Yellow Perch, we see some negativity during the late-2021 season into early 2022.

A common topic of discussion for Yellow Perch is in the context of eating and catching. Because of its declining populations in the lakes, several NCR states have regulations and daily harvest limitations on Yellow Perch. For example, Michigan has a daily harvest limit of 25, and no more than 5 of those should be 12 inches or greater in size. For Lake Erie, the daily limit is 50 fish [61]. There were frequent mentions of Lake Erie, reflecting the prevalence of this species in that lake. Additionally, one of the most frequent terms that showed up in mentions about Yellow Perch is the term ‘Walleye’, which likely reflects the close proximity these fish maintain to one another, allowing anglers to fish for both with ease.

We performed the Ljung-Box test for white noise and found evidence of autocorrelation in each time series, with the exception of Bluegill sentiment and Rainbow Trout. Dickey-Fuller unit root tests on non-differenced series revealed a stationary time series. With this evidence in mind, we observe short-memory autocorrelation; this is common when working with environmental series [62]. These results also motivate the use of Spearman rank correlations as a non-parametric approach robust to short-memory autocorrelation. Examining Spearman rank correlations (with associated P-values) between the mentions and net sentiment of species in Table 2 yields a few insights. Comparing mentions by species to mentions, all pairs reveal significant positive correlations of moderate strength, ranging from 0.23 to 0.63 (Bluegill to Largemouth Bass being the strongest). Sentiment to sentiment reveals no significant relationships. Comparing sentiment to mentions within species, we note that Walleye shows an inverse relationship (−.19, p = 0.005) and Bluegill is weakly positive (0.15, p = 0.03). Other relationships between mentions and sentiment are insignificant. This suggests discussions of these fish are homogeneous over time and that the overall tone of online media discussions about these fish (net sentiment) is not likely to be reacting simultaneously to shifts in volumes of mentions. This contrasts with some other topics in online and social media studies, where a surge in mentions corresponds to a decrease in sentiments [23]. As previously established, this may be due to differing latent features specific to the individual topics. Our results suggest fishing online discourse is community-driven, resembling the bird-watching communities studied by Ma et al. [28]. Comparing mentions and sentiment between species, we see a weak positive correlation between Bluegill Mentions and Largemouth Bass sentiment (0.16, p = 0.02), Walleye sentiment and Yellow Perch mentions show a weak negative correlation (−0.13, p = 0.04). Additionally, we find that Rainbow Trout and Yellow Perch sentiment are not significantly correlated with any variables studied. Otherwise, we lack a significant correlation between sentiment and mentions.

**Table 2:** Spearman rank correlation coefficients (with P-value) between mentions and net sentiment of species.

	Bluegill Mentions	Largemouth Bass Mentions	Walleye Mentions	Yellow Perch Mentions	Rainbow Trout Mentions	Bluegill Sentiment	Largemouth Bass Sentiment	Walleye Sentiment	Yellow Perch Sentiment
Bluegill Mentions	1.00								
Largemouth Bass Mentions	0.63***	1.00							
Walleye Mentions	0.61***	0.47***	1.00						
Yellow Perch Mentions	0.27***	0.23***	0.46***	1.00					
Rainbow Trout Mentions	0.37***	0.38***	0.55***	0.43***	1.00				
Bluegill Sentiment	0.15**	0.05	0.03	0.11	−0.02	1.00			
Largemouth Bass Sentiment	0.16*	0.12*	−0.05	−0.04	−0.08	0.13*	1.00		
Walleye Sentiment	−0.03	−0.05	−0.19***	−0.14***	0.06	0.03	−0.01	1.00	
Yellow Perch Sentiment	0.05	−0.04	−0.07	−0.02	−0.05	0.08	−0.02	0.01	1.00
Rainbow Trout Sentiment	0.06	0.05	0.12*	−0.08	0.07	0.02	0.02	0.07	−0.13*

Note that ***,**, and * denote statistical significance at the 1%, 5%, and 10% levels, respectively, and that statistically significant correlation coefficients (at the 10% or greater level) are in bold text.

Considering these findings in the context of other online media mention counts and sentiment studies of conservation and wildlife topics is challenging due to the relatively small body of literature dedicated to this topic and method. However, related studies have found contrasting results. One study of Rhinoceros conservation in online media found strong negative (and significant) correlations between mention counts and sentiment [63]. The lack of a relationship may be due to structural realities specific to fisheries [64]. Indeed, Rhinoceros populations face stressors far more existential than the stresses faced by the fishes studied here. As such, the Rhinoceros conservation movement may be dominated by a crisis mindset, whereas the online media regarding our fish species seems more in line with community building [28]. For example, a related study finds that online angler sentiment increased with season length and bag limit increases, which suggests that the sentiment online may be more related to specific (but not all) policies determined by wildlife managers [8].

Discussions of farming and wild sources for these fish are rare across all species. While each species averaged at least 100 mentions per week, mentions of farmed or wild fish averaged less than 10 per week in each species. This suggests that there was not considerable discussion about the source of these fish when news and social media users discuss these species. This could suggest that source differentiation is not salient in the minds of those who engage in online discourse. Studies of consumer perception of wild versus farmed fishes find varying levels of acceptance for farmed options, but farmed fish are generally viewed as the inferior product [65–67]. However, findings also suggest that consumers have difficulty differentiating between farmed and wild-caught fish [66]. On this note, it is possible that consumers and online commenters may have limited awareness of production practices and potential origins of the fish species studied, especially in cases where a species (e.g., Walleye) may be stocked or naturally reproducing. This may also have been a case where the terminology we searched for does not match the jargon commonly used to describe these fish in online settings, in which case we would have under-detected the true level of conversation on this topic. Alternative keyword strategies may improve upon this by expanding the count of terms that qualify. For future research, we suggest consideration of ‘fresh,’ ‘stocked,’ ‘hatchery,’ ‘aquaculture,’ ‘raised,’ and ‘caught’. These results highlight an important disconnect between public discussion and fishery production systems, with implications for how fisheries and aquaculture producers communicate with their stakeholders. Because of these possibilities, we refrain from concluding that the general population captured in this analysis is uninterested, but that discussions may not be framed around the production origin of these species. Future research could more directly assess consumer awareness through more traditional survey or experimental methods and expanded keyword strategies in online media listening.

Examining the relationship between nationwide US online media data and surface temperatures in the Great Lakes and St. Clair yields a few notable outcomes (Table 3). While most of the fishes net sentiment does not show significant correlations to any of the surface temperatures, Largemouth Bass net sentiment is the exception. In the case of Largemouth Bass, we see significance at the 1% level and weak-positive correlations (correlation coefficient between 0.21 and 0.23) with surface temperatures on each of the 6 Lakes studied (as well as the Great Lakes average).

**Table 3:** Spearman rank correlation coefficients between great lakes surface temperatures (in celsius) and US online media data on the fishes.

Sentiments						
	Superior	Michigan	Huron	Erie	Ontario	Saint Clair
**Total Sentiment**	0.16**	0.17**	0.16**	0.18***	0.17***	0.17***
**Bluegill Sentiment**	−0.06	−0.02	−0.03	0.00	−0.01	0.03
**Largemouth Bass Sentiment**	0.23***	0.28***	0.27***	0.31***	0.30***	0.30***
**Rainbow Trout Sentiment**	−0.03	0.01	0.00	0.03	0.02	0.03
**Walleye Sentiment**	0.01	0.01	0.02	0.01	0.01	0.01
**Yellow Perch Sentiment**	0.02	0.00	0.01	0.00	0.01	0.00
**Mentions**						
	**Superior**	**Michigan**	**Huron**	**Erie**	**Ontario**	**Saint Clair**
**Total Mentions**	0.06	0.18***	0.16**	0.25***	0.19***	0.31***
**Bluegill Mentions**	0.11	0.27***	0.25***	0.34***	0.29***	0.38***
**Largemouth Bass Mentions**	0.18***	0.28***	0.27***	0.33***	0.29***	0.37***
**Rainbow Trout Mentions**	−0.28***	−0.19***	−0.21***	−0.14**	−0.18***	−0.08
**Walleye Mentions**	−0.22***	−0.09	−0.11	0.00	−0.07	0.07
**Yellow Perch Mentions**	−0.40***	−0.36***	−0.37***	−0.34***	−0.36***	−0.30***

Note that ***,**,* denote statistical significance at the 1%, 5%, and 10% levels, respectively, and that statistically significant correlation coefficients (at the 10% or greater level) are in bold text.

We computed Spearman rank correlation coefficients on US nationwide mentions and sentiment against surface temperatures on the Great Lakes and Lake Saint Clair. Starting with Sentiments, the total collection of posts shows a weak but statistically significant positive correlation to surface temperatures on the Lakes tested. Bluegill, Rainbow Trout, Walleye, and Yellow Perch sentiments reveal no statistically significant correlation to surface temperatures. This may be due to the possibility that some of these mentions may not originate in regions around the Great Lakes examined here. Alternatively, the conversations may not be driven by ecological seasonal rhythms. Largemouth Bass sentiments show weak positive and statistically significant correlations to surface temperatures on each of the Lakes tested.

With mention counts, we observe that the total collection of mentions exhibits a weak but significant (p < 0.05) correlation to surface temperatures on each of the lakes, with the exception of Superior. Bluegill mentions follow this trend, showing weak positive correlations that are statistically significant with surface temperatures on each lake except Superior. Largemouth Bass mentions show weak positive and significant correlations to surface temperatures on each of the Lakes tested. Mentions of Rainbow Trout and Yellow Perch show negative weak to moderate correlations to surface temperatures on each Lake (with one exception – Rainbow Trout and Saint Clair). Walleye mentions show a weak negative correlation to surface temperatures only on Superior. We explore these relationships to evaluate them in the context of optimal water temperatures for the fishes. For example, research shows that Rainbow Trout and Walleye prefer water temperatures between 10° to 16°C, and 15° to 25°C, respectively [68,69]. Intuitively, mentions of Rainbow Trout demonstrate (weakly) negative correlations to surface temperatures, while Walleye shows a limited negative correlation in the case of Superior. Rainbow trout is a salmonid, and the direction of these correlations is broadly consistent with known species temperature preferences [12,13]. Contrasting this, some of the other fish prefer warmer temperatures; Largemouth Bass (20°C to 26°C), Bluegill (18°C to 27°C) [70–72]. Intuitively, these fishes show a positive correlation to mentions. This suggests directional co-movement between online discussion volume and surface temperatures. The results highlight the importance of careful consideration of catchability and temperature levels in fisheries management.

## Conclusions

Online media in news and social media about NCR fishes was identified and analyzed. We find that online media data is a useful medium to study angler sentiment on fishes that may be farmed and/or found in the wild. We find that the discourse captured is more community-driven than crisis-driven compared to other topics found in online media. We find limitations in discerning the species environment of origin (farmed or wild) to which the discussions pertain. Additionally, we find some degree of success in analyzing online media data specific to the fishes with readily available secondary data with which our primary data is loosely aligned. In this case, we employ secondary data on surface temperatures, which should impact recreation and commercial opportunities to fish in the wild.

We find that Walleye are the most commonly discussed of the species and that Yellow Perch are less commonly mentioned than the other fish studied in online media. Bluegill, Largemouth Bass, and Rainbow Trout saw similar four-year weekly average mentions counts of around 500. Every species except Bluegill saw its highest weekly average count of mentions in 2024. Bluegill mentions see seasonal spikes in summer, which is intuitive considering this fish is popular with anglers. Beyond our interest in how often people talk about the fishes, we investigate how people speak about the fishes in online media. We find “eating” is a common term with four of the fishes (Bluegill, Rainbow Trout, Walleye, Yellow Perch). This finding is consistent with related studies of online media, which find that the culinary interaction with livestock is a common topic of discussion in online media [41]. This may reflect that the community-building topics of these products extend beyond the recreational fishing context, into culinary topics. We also find “catching” is a common term for three of the fishes (Bluegill, Largemouth Bass, and Yellow Perch), although all of the species are commonly fished. When comparing the species total mentions to mentions that specifically included terms related to wild or farmed fish, we notice a drop-off; roughly 1% to 5% of total mentions about each of these species include information referencing wild or farmed fish. This suggests that the discussions are more general and that online discussion may not explicitly reflect the nuances of human interaction with these fish.

Further, we analyzed the relationship between these online media data pertaining to the fishes and surface temperatures on each of the Great Lakes and St. Clair, which connects Huron and Erie. Bearing in mind that online media discussions may not necessarily emerge in this same region, we find that the net sentiment scores for most of the species are not related to surface temperatures in the Lakes observed, with Largemouth Bass being the only exception. Effectively, we do not observe strong evidence that sentiment trends together with regional surface temperatures, presenting an opportunity for future research. Though, as discussed below, this could also reflect that mentions often originate outside the Great Lakes region or that conversation is not driven by ecological seasonality. Mentions of these fishes show more frequent and consistent significant correlations to surface temperatures compared to sentiment. The direction and strength of these correlations vary by species. We also observe some evidence of the preferred water temperature of these fish reflected in the mention counts. Analysis of correlations between species suggests that mention counts across all five species tend to move together over time, which is intuitive given the seasonal nature of fishing activity. The strongest correlation we observe occurs between Bluegill and Largemouth Bass (0.63, p < 0.001). This finding makes sense given their shared habitat and angler attention to them. These findings suggest that online discussion of these species is driven at least partially by fishing community discussion, beyond mere species-specific discussion. Contrasting this, sentiment-to-sentiment findings between species suggest these sentiments are uncorrelated. This may indicate that emotions on these topics do not spill over from one species to another.

This study and its findings, while exploratory in nature, suggest some potential directions for fisheries and wildlife managers at the State and Federal levels. We perform a proof of concept for online media studies at the intersection of recreational and commercial markets. Similar studies, with perhaps a narrowed scope, could supplement or reduce the cost of traditional creel surveys but not replace them; due to limitations (discussed later) in online media [64]. While creel surveys offer invaluable insights into fishing activity, they are resource-intensive and they are temporally limited. These contrast with online media listening, which can enable longer periods of study with fewer resources invested. Through continuous monitoring of wildlife discussions online, wildlife managers may be able to complement insights garnered from techniques like creel surveys. Namely, sustained declines in net sentiment (reflecting more negative postings relative to positive) could be a useful early indicator of shifts in public sentiment and attention, which would warrant closer and timelier field study. Our findings that Bluegill mentions peak seasonally with every summer are consistent with known angling patterns. Further, the correlation we observe between the Largemouth Bass sentiment and Great Lakes surface temperatures (though not observed in the other fishes) suggests this relationship may merit further investigation rather than indications of a general pattern seen across the fishes. Future work could build on baselines established here by empirically testing whether sustained, multi-week declines in net sentiment below species-specific baselines reliably precede documented shifts in angler behavior or fishery conditions. Limitations of this study include that we only collected English-speaking posts, which may underrepresent non-English-speaking communities, specifically those with ties to the selected species of fish studied. Quid is a proprietary commercial platform that assists researchers in more efficient collection of posts, in compliance with diverse terms and conditions between platforms, and in creating an anonymized, aggregated dataset. To our knowledge, there is no independent, peer-reviewed validation of Quid’s sentiment engine, a limitation shared by studies using comparable commercial social listening platforms [73,74]. Because of this, the authors manually review posts before collection (evenly distributed by year) to examine the accuracy of sentiment scoring. Where necessary, Quid enables refining of keyword classification rules, which helps us to partially address the issue. However, we cannot fully rule out the possibility of sentiment misclassification. As such, we advise interpretations of net sentiment as a broad indicator of the direction of public opinion rather than precise measurements [75]. Finally, the data in this paper reflect mentions of selected fish species from across the US, and these are compared to environmental conditions in the Great Lakes region. As such, results and evidence presented herein are exploratory evidence of associations between public sentiment toward fish species that may be found on the Great Lakes and regional environmental conditions. To this effect, we do not establish causal relationships.

## Supporting information

S1 Data Data. (XLSX)
